# Expression and Potential Biomarkers of Regulators for M7G RNA Modification in Gliomas

**DOI:** 10.3389/fneur.2022.886246

**Published:** 2022-05-09

**Authors:** Zhen Chen, Zhe Zhang, Wei Ding, Jie-hui Zhang, Zi-long Tan, Yu-ran Mei, Wei He, Xiao-jing Wang

**Affiliations:** ^1^Department of Neurosurgery, The Second Affiliated Hospital of Nanchang University, Nanchang, China; ^2^Yifeng County People's Hospital, Yichun City, China; ^3^Department of Neurology, The First Affiliated Hospital of Anhui Medical University, Hefei, China

**Keywords:** N7-methylguanosine, gliomas, epigenetics, prognostic signature, bioinformatics

## Abstract

Gliomas are the most frequent primary malignant brain tumors of the central nervous system, causing significant impairment and death. There is mounting evidence that N7 methylguanosine (m7G) RNA dysmethylation plays a significant role in the development and progression of cancer. However, the expression patterns and function of the m7G RNA methylation regulator in gliomas are yet unknown. The goal of this study was to examine the expression patterns of 31 critical regulators linked with m7G RNA methylation and their prognostic significance in gliomas. To begin, we systematically analyzed patient clinical and prognostic data and mRNA gene expression data from The Cancer Genome Atlas (TCGA) and the Gene Expression Omnibus (GEO) databases. We found that 17 key regulators of m7G RNA methylation showed significantly higher expression levels in gliomas. We then divided the sample into two subgroups by consensus clustering. Cluster 2 had a poorer prognosis than cluster 1 and was associated with a higher histological grade. In addition, cluster 2 was significantly enriched for cancer-related pathways. Based on this discovery, we developed a risk model involving three m7G methylation regulators. Patients were divided into high-risk and low-risk groups based on risk scores. Overall survival (OS) was significantly lower in the high-risk group than in the low-risk group. Further analysis showed that the risk score was an independent prognostic factor for gliomas.

## Introduction

Gliomas square measure the foremost frequent primary central systema nervosum tumors in humans, accounting for 30% of all primary brain tumors and 81% of malignant brain tumors (1). Due to their unique biology and clinical properties, gliomas are a highly varied group of brain tumors. Traditionally, gliomas subtypes and grades were determined primarily by histological characteristics. However, this classification technique does not adequately replicate the tumor's heterogeneity, is subject to subjectivity and disagreement among neuropathologists, falls short of precisely forecasting disease course, and cannot be used to guide treatment in a reliable manner (2). Despite advancements in surgical procedures and clinical regimens, high-grade gliomas therapy remains difficult and is associated with poor therapeutic effectiveness and overall survival (3). As a result, it is critical to develop innovative and trustworthy molecular signs for prognostic prediction in order to improve the dismal outcomes of patients with gliomas.

Currently, RNA methylation is a widespread post-transcriptional modification found in eukaryotes, prokaryotes (4, 5), and a few archaea (6). As with epigenetic modifications at the DNA and protein levels, these posttranscriptional RNA modifications, included N1-methyladenosine, N7-methylguanosine (m7G), 5-Methylcytidine (m5C), pseudouridine, N6-methyladenosine (m6A), and2-O-methylation (7), can be dynamically and reversibly regulated by specific enzymes (8). Among these, m7G, or guanosine methylation at position N7, is a highly conserved RNA modification found in tRNA, rRNA, mRNA 5′cap, and internal mRNA regions, where it plays a vital role in regulating RNA processing, metabolism, and function (9).

RNA modifications are required for the metabolism of RNA, the regulation of gene expression, and the development of human diseases such as cancer. Recent research demonstrates that various RNA modifications play critical roles in cancer. For instance, when overexpressed, METTL3, an m6A “writer” that modifies a large subset of messenger RNAs, is carcinogenic (10). Lan et al. demonstrated that KIAA1429 is significantly expressed in hepatocellular carcinoma and is linked with poor prognosis (11). Deposition of m5C by NSUN2 and mcm5s2U alteration at tRNA nucleotide position 34 play crucial roles in cancer. Increased levels of m1A, which are linked to hTrm6p/hTrm61p, were found to be linked to an increased risk of urinary bladder cancer (12). In eukaryotes, METTL1 and WDR4 form a methyltransferase complex that catalyzes the m7G modification of tRNAs (13). Dai et al. discovered that silencing METTL1 or WDR4 in human intrahepatic cholangiocarcinoma cell lines resulted in the loss of typical malignant transformation markers such as stalled cell proliferation, increased apoptosis, reduced colony formation, and migration, and decreased tumor formation in xenografted mice (14). These studies demonstrate that abnormal RNA modifications can have an effect on tumor initiation and progression. Additionally, methylation of RNA has been shown to have a key role in gliomas. Recent studies have reported that m6A RNA methylation regulators are critical in maintaining glioma stem cells and radioresistance and have a potential role in prognostic and therapeutic strategy development (15). However, there is a dearth of thorough studies examining the expression of m7G RNA methylation regulators in gliomas, their role in malignant progression, and their prognostic relevance.

The expression of 31 major regulators of m7G RNA modification in gliomas was assessed in this work using transcriptome data from The Cancer Genome Atlas (TCGA) database. Additionally, by using consensus clustering, glioma patients were classified into two clusters based on the expression of regulators of m7G RNA modification, and the two clusters had dramatically different prognoses. Additionally, a signature incorporating three selected m7G RNA methylation regulators was developed to stratify glioma prognosis.

## Materials and Methods

### Data Collection

RNA-sequencing transcriptome data and clinical data on patients with glioma were extracted from the TCGA database (https://portal.gdc.cancer.gov/; until January 2022). A total of 698 glioma patients and 5 normal surrounding tumor tissues were used for further investigation. We downloaded 31 m7G methylation-related genes from the Gene Set Enrichment Analysis (GSEA) database (http://www.gsea-msigdb.org/gsea/index.jsp), including DCP2, DCPS, NUDT1, NUDT10, NUDT11, NUDT16, NUDT16L1, NUDT3, NUDT4, NUDT4B, NUDT5, NUDT7, AGO2, CYFIP1, CYFIP2, EIF4E, EIF4E1B, EIF4E2, EIF4E3, GEMIN5, LARP1, NCBP1, NCBP2, NCBP3, EIF3D, EIF4A1, EIF4G3, IFIT5, LSM1, NCBP2L, and SNUPN (collect and download from GSEA). The expression data for these 31 genes were retrieved for further investigation from the TCGA gliomas group. External validation was performed using an independent cohort (GSE4412) encompassing glioma samples with associated gene expression and survival data collected from the Gene Expression Omnibus (GEO) database (http://www.ncbi.nlm.nih.gov/geo).

### Bioinformatics Analysis

The Wilcoxon test was utilized to identify genes encoding m7G RNA methylation regulators that were differentially expressed across gliomas and normal tissues. The following criteria were used to determine significance: A false discovery rate (FDR) of <0.05 and an absolute Fold change (FC) >1.5 are required. The expression of these m7G-related genes was then visualized using a vioplot in 698 Glioma patients and five normal neighboring samples. We used Spearman correlation analysis in R (https://www.r-project.org/, version R 4.1.2) to determine the relationship between m7G RNA methylation regulators. The packages of “limma,” “ggplot2,” “pheatmap” and “corrplot” were used in these analyses.

To evaluate the relation between the expression of m7G RNA methylation regulators and glioma prognosis, the glioma tissues were grouped into two distinct subgroups exploitation the R package “ConsensusClusterPlus.” Then, principal component analysis (PCA) was used to validate the categorization findings using the “ggplot2” and “limma” packages.

To assess survival between subgroups, a survival curve was produced using the Kaplan–Meier analysis log-rank test. The Chi-square tests were used to compare the differences in clinical parameters between the two clusters. Functional annotation of differentially expressed genes in two clusters using Kyoto Encyclopedia of Genes and Genomes (KEGG) and Gene Ontology (GO) analysis. The link between m7G-related genes and overall survival (OS) was evaluated using univariate Cox regression models. Subsequently, to minimize overfitting, To eliminate highly associated genes, we utilized the “glment” package to perform least absolute shrinkage and selection operator (LASSO) Cox regression. Finally, a risk profile consisting of three m7G regulatory genes was found. To calculate a risk score, we multiplied the gene expression by the LASSO Cox regression coefficient. Patients with glioma were then categorized into low- or high-risk groups based on median risk scores. Kaplan–Meier analysis was performed using the “Survival” software. The accuracy of the model for prognostic prediction was determined using receiver operating characteristic (ROC) curves. Clinicopathological characteristics were compared between low- and high-risk groups using the Chi-square test and displayed using a heatmap.

We utilized the GSE4412 dataset from GEO to evaluate the predictive usefulness of this three-m7G-regulatory-gene risk profile. We adopted this cutoff criterion to categorize patients as low- or high-risk. Following that, the prognostic value was determined using Kaplan–Meier survival analysis and receiver operating characteristic curve analysis. Moreover, to use the “rms” package, we used the risk score of three m7G-regulatory genes to predict survival in patients with glioma. All of the R packages listed before were downloaded from http://www.bioconductor.org.

### Statistical Analysis

All statistical analyses were conducted using R software (version 4.1.2).

## Results

### Identification of Glioma-Specific m7G RNA Modification Regulators

**Figure 1:**
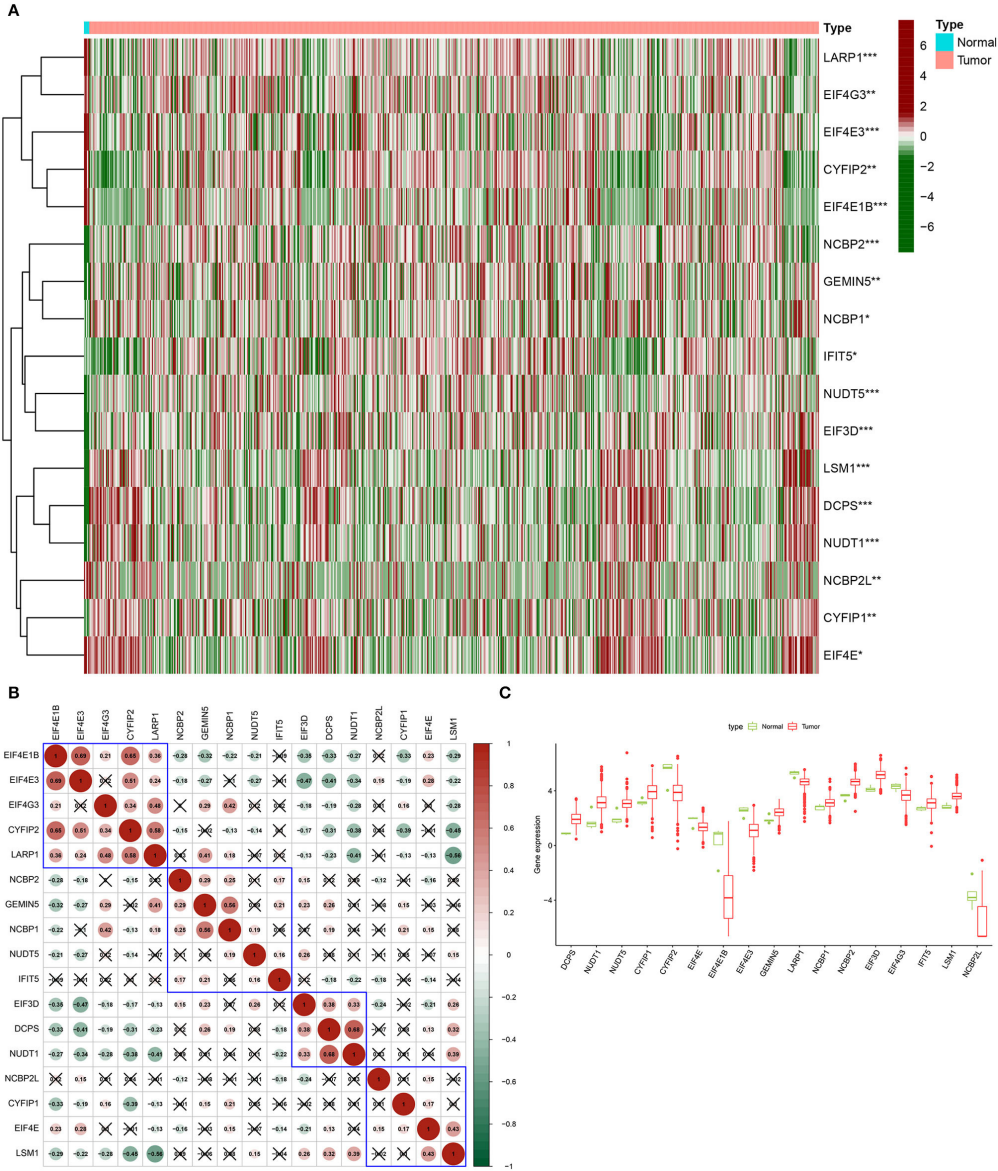
Expression of m7G methylation regulators in glioma tissues and normal tissues. **(A)** Heat map showing 17 m7G RNA methylation regulators expressed at different levels between the two samples (Red for high expression level, green for low expression level. *p < 0.05, **p < 0.01, ***p < 0.001). **(B)** Spearman correlation analysis of 17 m7G RNA methylation regulators in gliomas (Red for positive correlation, green for negative correlation). **(C)** Vioplot visualizes the expression levels of 17 m7G RNA methylation regulators in two samples.

The differentially expressed 17 m7G regulatory genes were analyzed in Glioma (*n* = 698) and surrounding tissues (*n* = 5). The heatmap demonstrated unequivocally that the majority of these m7G regulatory genes were differently expressed across glioma and normal tissues (Figure 1A). NUDT1, DCPS, NUDT5, EIF3D, NCBP2, CYFIP1, LSM1, GEMIN5, IFIT5, NCBP1, EIF4E, EIF4G3, LARP1, EIF4E3, CYFIP2, NCBP2L, and EIF4E1B expression levels were significantly different between the glioma group and normal group. The relevant heat map and box-plot of these differential genes are shown, respectively, in Figures 1B,C.

### Clusters of Glioma Patients Identified by Consensus Clustering Based on m7G RNA Modification Regulators

**Figure 2:**
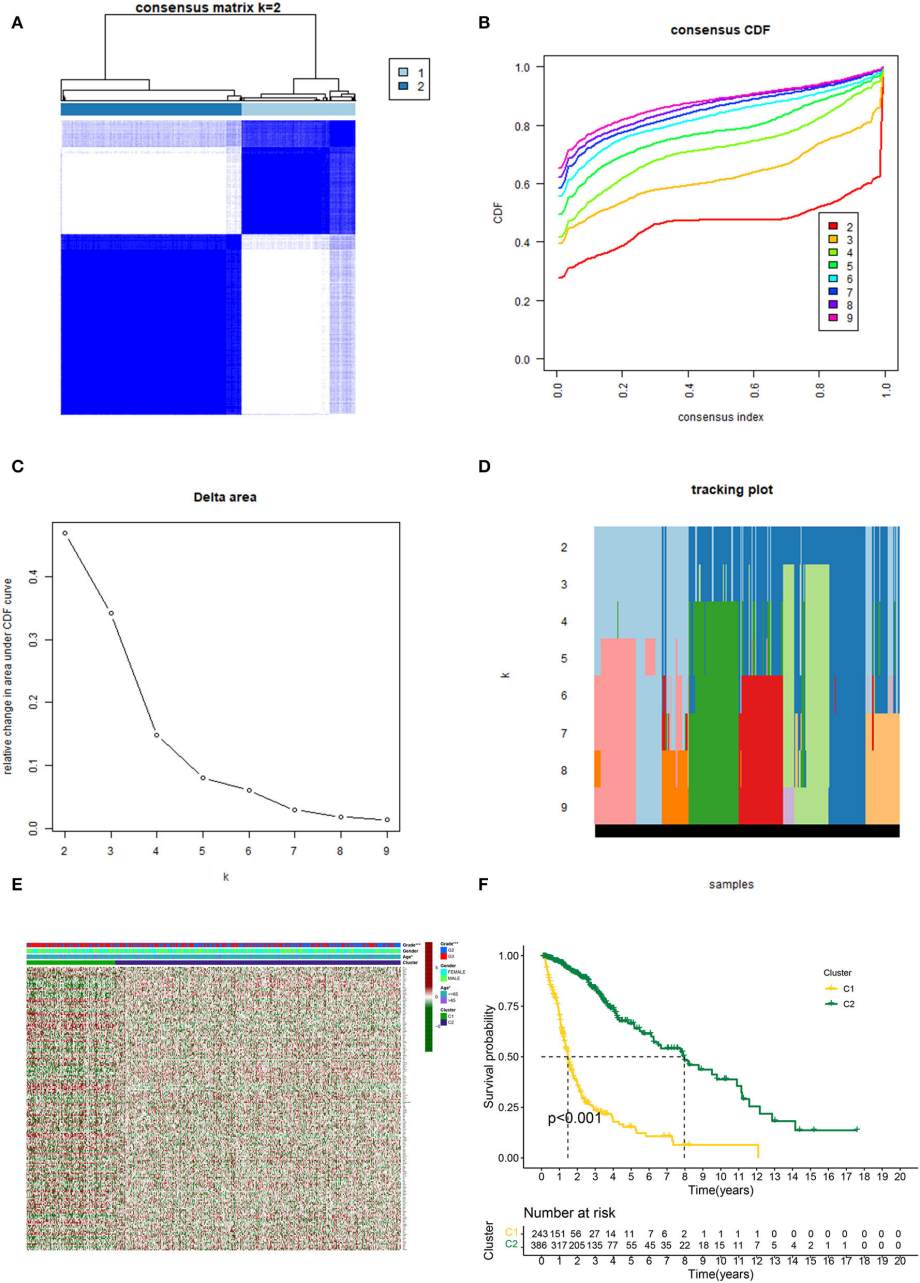
Consistent cluster analysis of gliomas. **(A)** Consensus clustering matrix with *k* = 2. **(B)** Cumulative distribution function (CDF) for consistent clustering at *k* = 2–9. **(C)** For *k* = 2–9, the relative change in the area under the CDF curve. **(D)** Sample distribution for *k* = 2–9. **(E)** Heatmap and clinicopathologic characteristics of the two clusters established by the consensus expression of the m7G RNA methylation regulators (Red for high expression level, green for low expression level). **(F)** Comparison of Kaplan–Meier OS rates for overall survival (OS) curves in the two clusters (*p < 0.05, ***p < 0.001).

**Figure 3:**
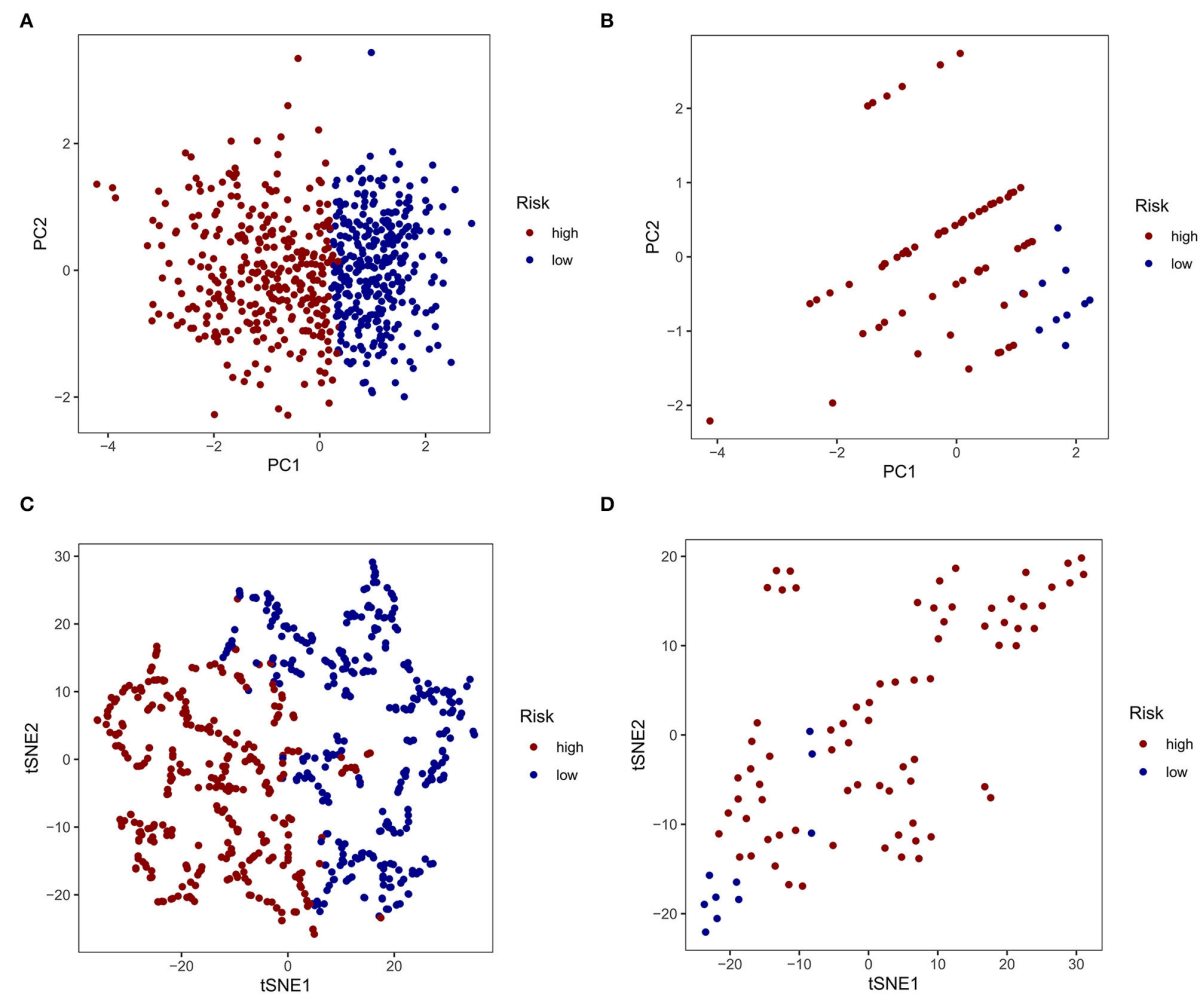
Principal component analysis (PCA) and t-distributed stochastic neighbor embedding (t-SNE) analysis. **(A, C)** PCA and t-SNE analysis of Clusters 1 and 2 from the TCGA database. **(B, D)** PCA and t-SNE analysis of Clusters 1 and 2 from the GEO database.

Based on the similarity of expression of the m7G RNA methylation regulators, *k* = 2 seemed to be the best cluster to divide these samples into two clusters, i.e., clusters 1 and 2 (Figures 2A–D). Furthermore, the expression of most m7G RNA modification regulators in cluster 1 was greater than in cluster 2 (Figure 2E). Cluster 1 was strongly linked with high grade (*p* < 0.001) and high age (*p* < 0.05). However, gender was not shown to be a major factor (Figure 2E). Thus, the consensus clustering results suggested a strong correlation between the expression pattern of m7G RNA modification regulators and the malignant state of glioma. As a consequence, cluster 2 has a significantly lower OS than cluster 1 (*p* < 0.001) (Figure 2F). To validate the clustering results, we used PCA and t-SNE to examine these two clusters further. The PCA and t-SNE plots revealed a significant difference between clusters 1 and 2 (train: Figures 3A,C; test: Figures 3B,D).

**Figure 4:**
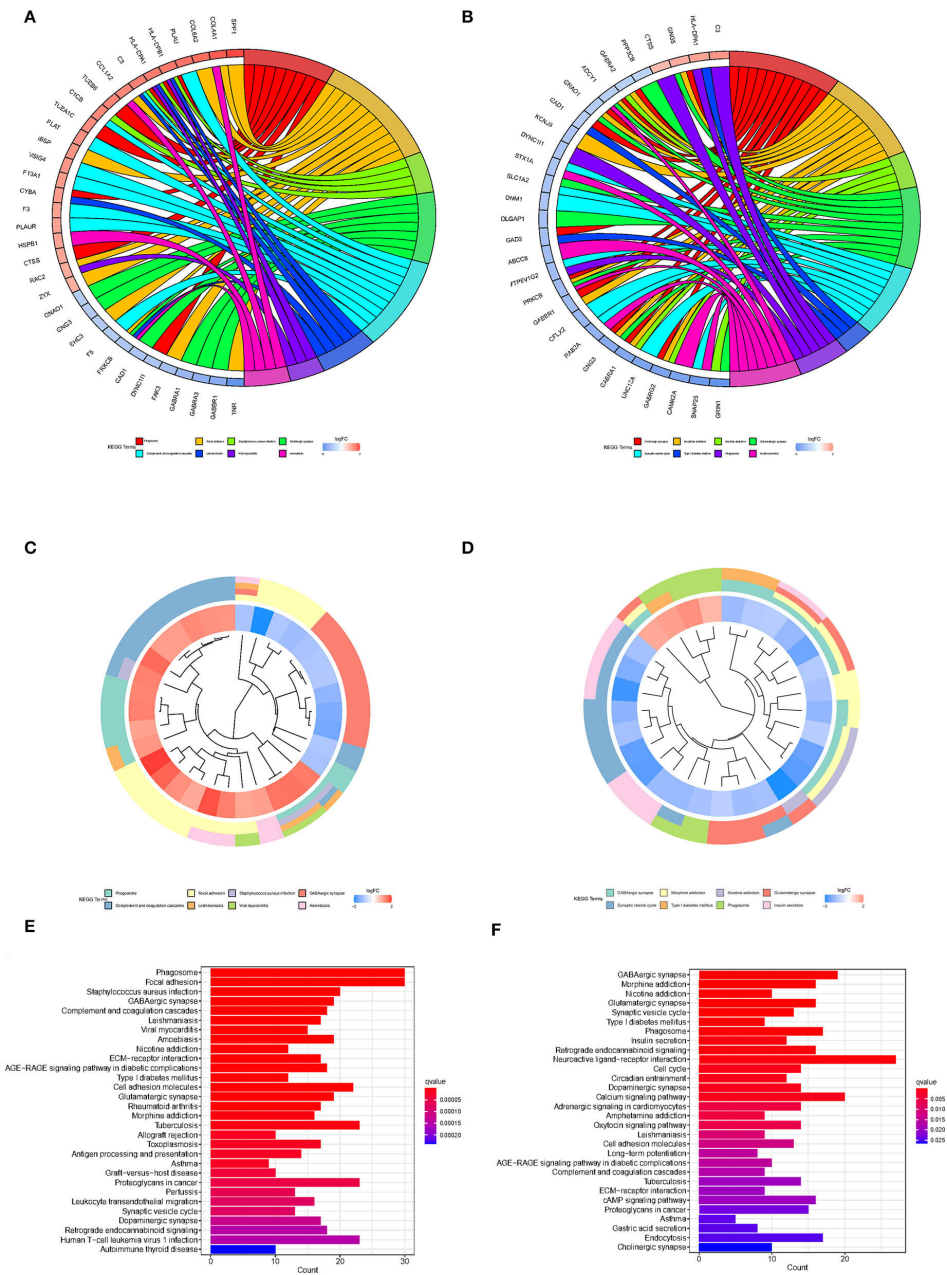
Analyses of differentially expressed genes between two clusters using the Kyoto Encyclopedia of Genes and Genomes (KEEG). **(A, C, E)** KEGG analysis for functional annotation of differentially expressed genes in two clusters of the TCGA database. **(B, D, F)** KEGG analysis for functional annotation of differentially expressed genes in two clusters of the GSE4412 data sets.

**Figure 5:**
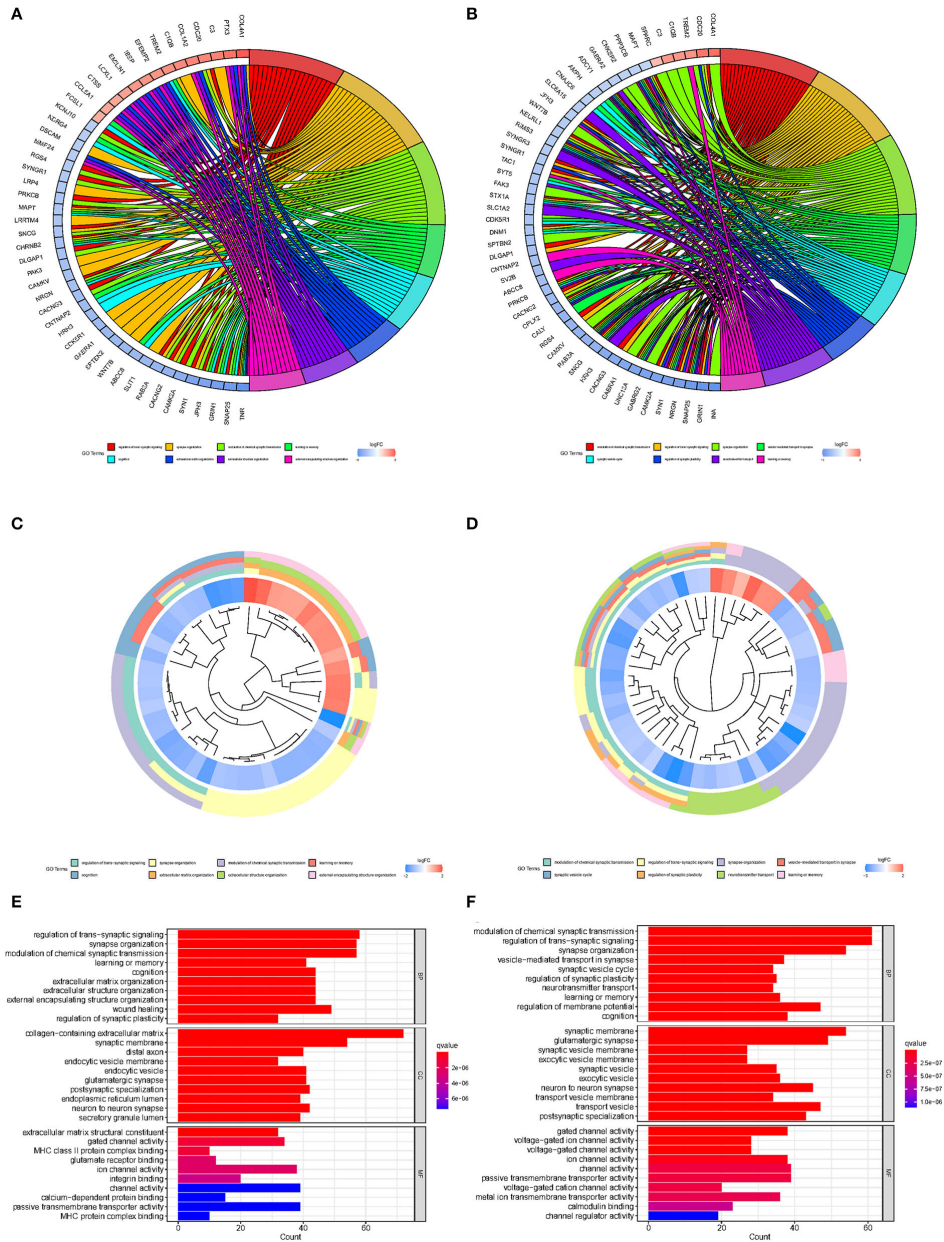
Analyses of differentially expressed genes between two clusters using the Gene ontology (GO). **(A, C, E)** GO analysis for functional annotation of differentially expressed genes in two clusters of the TCGA database. **(B, D, F)** GO analysis for functional annotation of differentially expressed genes in two clusters of the GSE4412 datasets.

To explore the biological processes associated with m7G RNA methylation regulators in gliomas, functional annotation of differentially expressed genes in clusters 1 and 2 was performed using GO and KEGG analysis. In the TCGA group, according to the KEGG analysis, the differential genes were primarily enriched in “Phagosome,” “Focal adhesion,” “Staphylococcus aureus infection,” “GABAergic synapse,” “Complement and coagulation cascades Leishmaniasis,” “Viral myocarditis,” “Amoebiasis,” “Nicotine addiction,” “ECM-receptor interaction,” “AGE-RAGE signaling pathway in diabetic complications Type I diabetes mellitus” (Figures 4A,C,E). In the GSE4412 group, the KEGG analysis revealed that the differential genes were primarily enriched in “GABAergic synapse,” “Morphine addiction,” “Nicotine addiction,” “Glutamatergic synapse,” “Synaptic vesicle cycle,” “Type I diabetes mellitus,” “Phagosome,” “Insulin secretion,” “Retrograde endocannabinoid signaling,” “Neuroactive ligand-receptor interaction” (Figures 4B,D,F). In the TCGA group, the GO analysis revealed that the differential genes were primarily enriched in “Regulation of trans-synaptic signaling,” “Synapse organization,” “Modulation of chemical synaptic transmission,” “Learning or memory,” “Cognition,” “Extracellular matrix organization,” “Extracellular structure organization,” “External encapsulating structure organization,” “Wound healing,” “Regulation of synaptic plasticity” (Figures 5A,C,E). In the GSE4412 group, the GO analysis revealed that the differential genes were primarily enriched in “Modulation of chemical synaptic transmission,” “Regulation of trans–synaptic signaling,” “Synapse organization,” “Vesicle–mediated transport in synapse,” “Synaptic vesicle cycle,” “Regulation of synaptic plasticity,” “Neurotransmitter transport,” “Learning or memory,” “Regulation of membrane potential,” “Cognition” (Figures 5B,D,F).

### Development of a Predictive Risk Model Based on m7G Regulator Gene Expression Profiles and Validation Using the GEO Database

**Figure 6:**
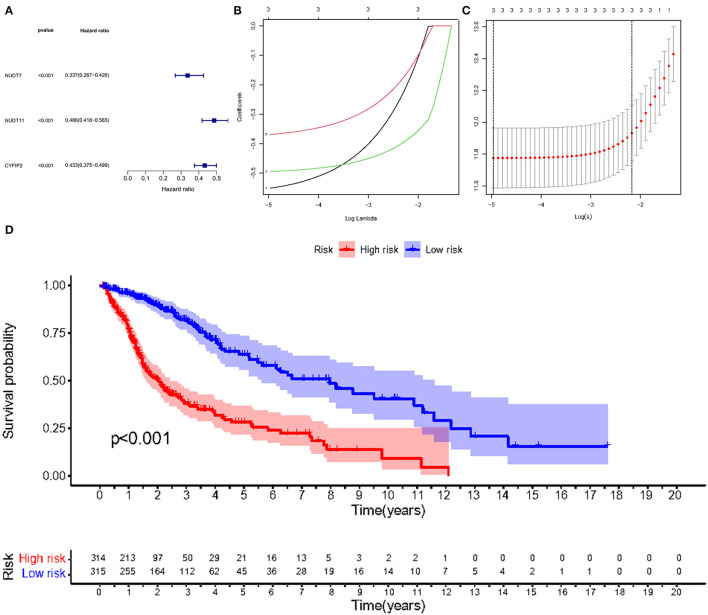
Risk signature with three m7G RNA methylation regulators. **(A)** Univariate Cox regression analysis of the m7G RNA methylation regulators. **(B, C)** LASSO regression analysis was used to calculate the coefficients of the three m7G RNA methylation regulators. **(D)** Glioma patients were divided into low- and high-risk groups based on the median risk score.

**Figure 7:**
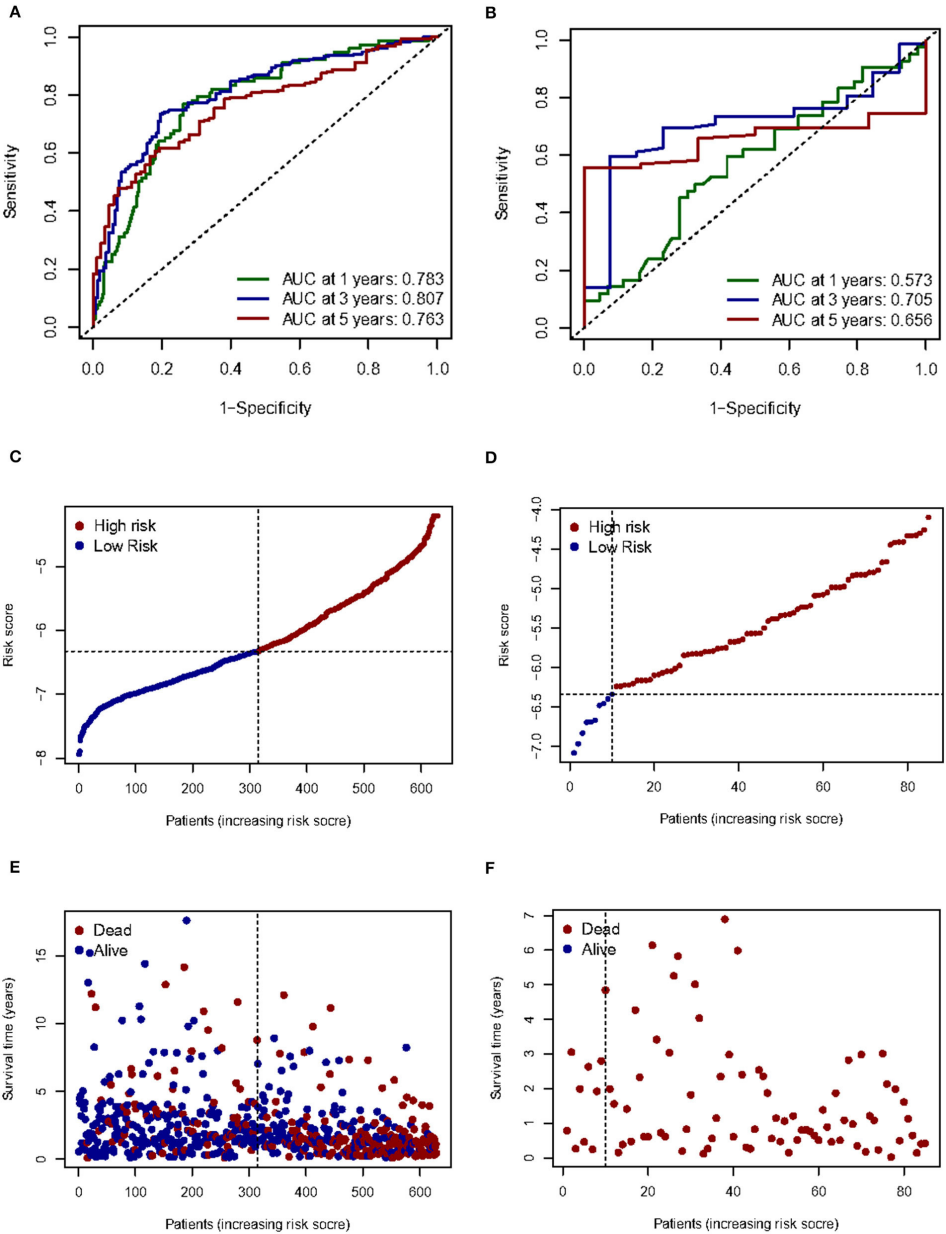
Prognostic value of three m7G RNA methylation regulators. **(A, B)** In the TCGA and GEO databases, the receiver operating characteristic (ROC) curves demonstrate the risk scores' predictive ability for patient survival at 1, 3, and 5 years. **(C,D)** Distribution of risk scores for glioma patients. **(E,F)** Survival status of each patient.

**Figure 8:**
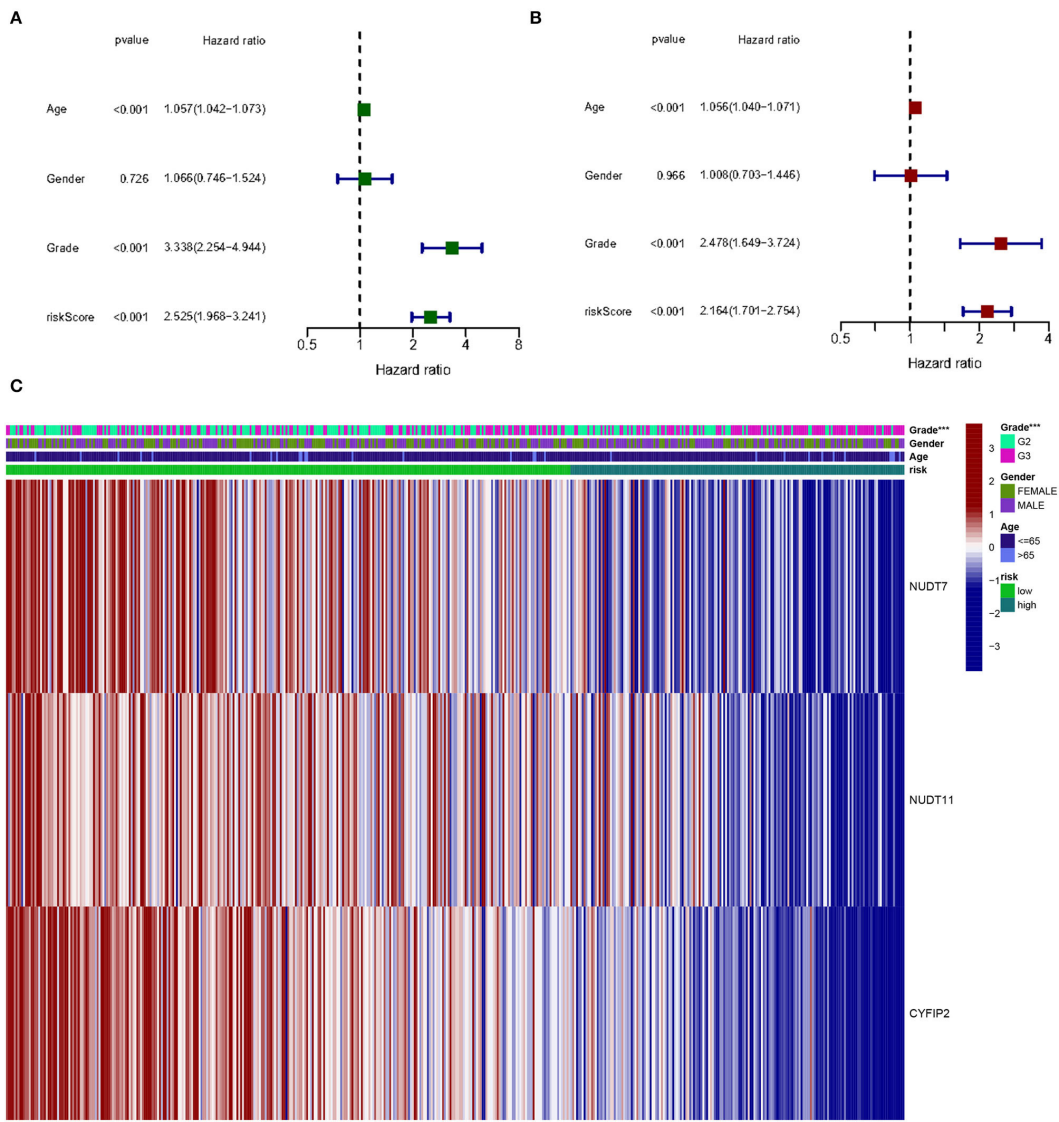
The effect of a risk score on the prognosis of patients with gliomas. **(A)** Univariate Cox regression analysis of three-gene risk score, age, and grade. **(B)** Multivariate Cox regression analysis of three-gene risk score, age, and grade (***p < 0.001). **(C)** Heat map shows the distribution of the expression of three m7G RNA-modifying regulators and clinicopathological features in high- and low-risk populations.

Given the high correlation between m7G RNA methylation regulators and glioma patient prognosis. Combined with the results of the TCGA and GSE4412 datasets, three genes (NUDT7, NUDT11, CYFIP2) were chosen for the development of the risk model (Figure 6A), along with the related LASSO method coefficients (Figures 6B,C). The univariate Cox regression analysis of these genes showed that NUDT7, NUDT11, and CYFIP2 are all protective factors with a hazard ratio (HR) of less than 1 for the OS of glioma patients. To evaluate the prognostic value of this three-gene signature model, glioma patients were separated into low- and high-risk groups based on their median risk score. Survival analysis revealed that patients with a high-risk score had a lower OS than patients with a low-risk score (Figure 6D). The ROC curve analysis showed that the AUC for 1-, 3-, and 5-year OS was 0.783, 0.807, and 0.763, respectively. The above results indicated the risk model has good discrimination for the outcome of patients with glioma (Figure 7A). Additionally, as illustrated in Figure 7C, the risk score distribution of glioma patients was displayed. The survival status of each patient was represented by a dot plot (Figure 7E). A heatmap was created to show the expression of three prognostic genes in high- and low-risk groups (Figure 8C). Above the heatmap, clinical importance was also shown. To verify the predictive discrimination of the gene signature; we used the GEO data (GSE4412) to assess the prognostic utility of the three-gene signature in new files for survival prediction. The area under the curve (AUC) for the ROC curves for 1, 3, and 5 year OS was 0.573, 0.705, and 0.656, respectively (Figure 7B). Additionally, as illustrated in Figure 7D, the risk score distribution of glioma patients was displayed. The survival status of each patient was represented by a dot plot in Figure 7F.

Univariate and multivariate Cox regression analysis to determine the prognostic value in m7G methylation regulator based on clinical information on gliomas in the TCGA database. By univariate analysis, trigene risk score, age, and grading were all significantly associated with OS in patients with glioma (Figure 8A). To determine whether the trigene risk score was an independent prognostic factor for glioma independent of other clinical factors, these factors were included in a multivariate Cox regression analysis, which showed that all three gene risk scores, age, and grade were independently associated with OS in glioma patients (Figure 8B).

## Discussion

Despite recent breakthroughs in glioma treatment, effective glioma therapy remains an unmet medical need, as current treatments, which often include a mix of surgical intervention (tumor excision), radiotherapy, and chemotherapy, do not achieve satisfying results. The prognosis for patients remains poor, with a median OS of approximately 15 months for those with the most aggressive grade IV glioblastomas (16). According to the WHO, cellularity, mitotic activity, nuclear atypia, vascularity, and necrosis are used to grade (17). This classification method has some limitations, including a lack of consistency, which results in a high rate of inter- and even intra-observer variability, and a lack of prognostic precision (18, 19). As a result, there is an urgent need to understand the molecular mechanisms driving glioma formation.

Traditional molecular techniques, which have mostly focused on structural changes in genes such as point mutations, gene deletions, and rearrangements, have provided vital insights into the glioma-genesis process as well as the diagnostic, prognostic, and predictive power of these tumors (20). For the past few years, understanding the role of epigenetic modifications in the development of human tumors has added critical pieces to the gliomagenesis puzzle and will result in the discovery of novel prognostic biomarkers and therapeutic techniques. DNA methylation and histone alterations are two of the most well-studied epigenetic changes in gliomas, and they are both associated with an increased risk of cancer (21, 22). Recently, RNA methylation has been found to be another way to control your DNA. RNA has been revealed to have over 170 chemical modifications, which play important roles in many cellular processes (23, 24). Among them, m7G, the most prevalent RNA cap modification, is co-transcribed with the 5′ cap during the earliest phases of transcription and prior to subsequent RNA processing processes (25). The m7G capping is a positively charged RNA modification that is required for gene expression, protein synthesis, and transcript stabilization. It has been discovered that m7G cap alteration can influence practically every part of the mRNA life cycle (26). The m7G RNA alteration was also discovered in tRNA (27) and rRNA (28), and its presence has been linked to a variety of illnesses. In humans, for example, a mutation in the methyltransferase complex WDR4 has been associated with primordial dwarfism, a condition marked by facial dysmorphism, brain malformation, and severe encephalopathy with convulsions (29). Because of the widespread use of RNA-seq and microarray techniques, which offer significant potential for the development of innovative diagnostics, risk score methods based on multiple gene signatures are increasingly being utilized to forecast human cancer prognosis (30). In the current investigation, we found that the expression of regulators of m7G RNA methylation was strongly associated with the malignancy and prognosis of gliomas. Using three m7G RNA modification regulators, we produced a predictive signature. Fortunately, the risk score was capable of independently predicting the fate of glioma patients. As a result, the risk signature identified in this study may aid clinicians in performing more accurate customized survival forecasts.

Previous research has established that the expression levels of the METTL1 and WDR4 components of the tRNA m7G methyltransferase complex are dramatically increased in human cancer samples and are negatively linked with patient prognosis. For example, Yang et al. found that overexpression of METTL1 inhibited colon cancer progression by regulating the let-7e miRNA/high mobility group AT-hook 2 axis in a m7G dependent manner (31). Chen, Z. et al. found that m7G tRNA modification and its catalase METTL1 and WDR4 proteins were significantly upregulated in hepatocellular carcinoma and inversely correlated with patient survival (32). Orellana et al. established a link between higher METTL1/WDR4 complex expression and elevated m7G tRNA levels and malignancy and poor survival in a variety of human malignancies, including glioblastomas, breast tumors, and acute myelogenous leukemias, among others (33), m7G methylation can selectively boost the translation of specific cell cycle regulating and carcinogenic mRNAs that are enriched in corresponding m7G-tRNA cognate codons, thereby buffering against ribosome stopping, which can result in translation inhibition *via* ribosome collisions (34). However, no research on m7G methylation in gliomas has been conducted. In this investigation, we compared the expression of all m7G RNA methylation regulators in glioma and normal tissues. The results indicated that tumor tissues expressed considerably more m7G regulatory genes than normal tissues. Additionally, we divided patients with glioma into subgroups based on their gene expression profiles. The consensus clustering results indicated a strong correlation between the expression pattern of m7G RNA methylation regulators and the gliomas malignancy. Based on previous findings, the reason may be related to the up-or down-regulation of specific m7G RNA methylation regulators associated with mis-regulated RNAs in tumors.

The m7G RNA methylation regulators were also linked to the biological processes and signaling pathways involved in the malignant development of gliomas, according to this study. According to current research, the main biological processes and signaling pathways associated with m7G methylation in cancer are RNA metabolism, embryonic stem cell self-renewal and differentiation (13), tumor immunity (35), vascular development (36), FGF/TGF-β/Wnt pathway (37), WNT/β-catenin pathway (38), MAPK/ERK pathway (39), and so on. Here, we conducted KEGG and GO analysis on differentially expressed genes. The KEGG analysis revealed that the differential genes were primarily enriched in “Phagosome,” “Focal adhesion,” “*Staphylococcus aureus* infection,” “GABAergic synapse,” “Complement and coagulation cascades Leishmaniasis,” “Viral myocarditis,” “Amoebiasis,” “Nicotine addiction,” “ECM-receptor interaction,” “AGE-RAGE signaling pathway in diabetic complications Type I diabetes mellitus,” “Morphine addiction,” “Glutamatergic synapse,” “Synaptic vesicle cycle,” “Type I diabetes mellitus,” “Insulin secretion,” “Retrograde endocannabinoid signaling,” “Neuroactive ligand-receptor interaction”. The GO analysis revealed that the differential genes were primarily enriched in “Regulation of trans-synaptic signaling,” “Synapse organization,” “Modulation of chemical synaptic transmission,” “Learning or memory,” “Cognition,” “Extracellular matrix organization,” “Extracellular structure organization,” “External encapsulating structure organization,” “Wound healing,” “Regulation of synaptic plasticity,” “Vesicle-mediated transport in synapse,” “Synaptic vesicle cycle,” “Neurotransmitter transport,” “Regulation of membrane potential.” According to the data collected, we discovered that the expression of m7G RNA methylation regulators in gliomas is associated significantly with biological processes such as extracellular matrix formation, response to hypoxia, inflammatory response, synaptic vesicle regulation, and others, as well as signaling pathways such as AGE-RAGE signaling pathway, ECM receptor interaction, and others.

To further investigate the effect of m7G RNA methylation regulators on glioma prognosis. After performing Cox univariate and LASSO Cox regression analyses, we selected three genes (NUDT7, NUDT11, and CYFIP2) to construct a risk score model that stratified glioma patients into low- and high-risk groups. The results showed that NUDT7, NUDT11, and CYFIP2 were all protective factors for OS in glioma patients. Survival analysis revealed that patients with high-risk scores had worse OS. Finally, we established a prognosis model consisting of these three m7G RNA methylation regulators. Cox regression analysis showed that the risk score can independently predict the prognosis of gliomas. These findings suggest that NUDT7, NUDT11, and CYFIP2 are promising gliomas prognostic biomarkers. Notably, the expression and potential role of NUDT7, NUDT11, and CYFIP2 in cancer are becoming increasingly important. NUDT7 and NUDT11 are both hydrolases that belong to the peroxisomal nudix family of enzymes (40, 41). Mutations of NUDT7 have been described in colorectal cancer, inhibition of Nudt7 may contribute to the progression of Kras ^G12D^ colorectal cancer through upregulation of Wnt/β-catenin signaling and palmitic acid accumulation (42). Genetic and functional analyses showed that when NUDT11 was inhibited, colony formation of tumor-associated cell phenotypes was significantly reduced and their proliferation/survival capacity was compromised (43). Therefore, genetic alterations in m7G regulatory factors may contribute to the pathogenesis of glioma in conjunction with dysregulation of peroxisome lipid metabolism. CYFIP2, a member of the cytoplasmic FMR1-interacting protein family (44). It has been reported that inhibition of CYFIP2 increased cell growth and resistance to chemotherapy in gastric cancer through activating the Akt signal (45). In addition, Saller et al. reported CYFIP2 as a candidate p53 target gene in H1299 lung cancer cells (46), and Jackson et al. verified in DLD1 colorectal adenocarcinoma cells that CYFIP2 can act as a p53-inducible gene to promote apoptosis in colorectal adenocarcinoma cells (47). Also, Tong et al. found that CYFIP2 expression was decreased in clear cell renal cell carcinoma and was associated with poorer clinicopathological parameters of clear cell renal cell carcinoma patients (48). CYFIP2 activates the cell death program to inhibit the proliferation of tumor cells, indicating that CYFIP2 is a potent tumor suppressor. In this study, we demonstrate for the first time that the risk scores of NUDT7, NUDT11, and CYFIP2 can be used as independent prognostic factors for gliomas.

Nevertheless, we acknowledge several limitations in this study worth mentioning. Since our data are derived from the TCGA and GEO databases, further experimental evidence is required to corroborate our findings. And the sample size for the normal and tumor groups varied significantly, which may impair the credibility of the results. In addition, the mechanisms underlying the aberrant expression and up- or down-regulation of m7G RNA methylation regulator in gliomas require further studies to validate.

In conclusion, our study demonstrates the expression of m7G RNA methylation regulator in gliomas and its association with clinical characteristics. Notably, prognostic models generated by three m7G methylation-regulated gene risk scores independently predicted the prognosis of glioma patients. These findings provide a new insight into the underlying mechanism of m7G modification in tumorigenesis and the development of gliomas.

## Data Availability Statement

The datasets presented in this study can be found in online repositories. The names of the repository/repositories and accession number(s) can be found in the article/Supplementary Material.

## Ethics Statement

Ethical review and approval was not required for the study on human participants in accordance with the local legislation and institutional requirements. Written informed consent from the patients/participants or patients/participants' legal guardian/next of kin was not required to participate in this study in accordance with the national legislation and the institutional requirements.

## Author Contributions

ZC, ZZ, WH, and X-jW conceived and designed the experiments. WD, J-hZ, and Z-lT collected the data. ZC and ZZ analyzed the data. WH and X-jW wrote the article. Z-lT and Y-rM conducted quality control on the articles and guided the submission. All authors read and approved the final manuscript.

## Funding

This study was supported by the Project of Youth Project of Jiang xi Provincial Department of Education (GJJ200244).

## Conflict of Interest

The authors declare that the research was conducted in the absence of any commercial or financial relationships that could be construed as a potential conflict of interest.

## Publisher's Note

All claims expressed in this article are solely those of the authors and do not necessarily represent those of their affiliated organizations, or those of the publisher, the editors and the reviewers. Any product that may be evaluated in this article, or claim that may be made by its manufacturer, is not guaranteed or endorsed by the publisher.
